# Powerful Haplotype-Based Hardy-Weinberg Equilibrium Tests for Tightly Linked Loci

**DOI:** 10.1371/journal.pone.0077399

**Published:** 2013-10-22

**Authors:** Wei-Gao Mao, Hai-Qiang He, Yan Xu, Ping-Yan Chen, Ji-Yuan Zhou

**Affiliations:** Department of Biostatistics, School of Public Health and Tropical Medicine, Southern Medical University, Guangzhou, Guangdong, China; University of Sydney, Australia

## Abstract

Recently, there have been many case-control studies proposed to test for association between haplotypes and disease, which require the Hardy-Weinberg equilibrium (HWE) assumption of haplotype frequencies. As such, haplotype inference of unphased genotypes and development of haplotype-based HWE tests are crucial prior to fine mapping. The goodness-of-fit test is a frequently-used method to test for HWE for multiple tightly-linked loci. However, its degrees of freedom dramatically increase with the increase of the number of loci, which may lack the test power. Therefore, in this paper, to improve the test power for haplotype-based HWE, we first write out two likelihood functions of the observed data based on the Niu's model (NM) and inbreeding model (IM), respectively, which can cause the departure from HWE. Then, we use two expectation-maximization algorithms and one expectation-conditional-maximization algorithm to estimate the model parameters under the HWE, IM and NM models, respectively. Finally, we propose the likelihood ratio tests LRT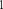 and LRT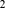 for haplotype-based HWE under the NM and IM models, respectively. We simulate the HWE, Niu's, inbreeding and population stratification models to assess the validity and compare the performance of these two LRT tests. The simulation results show that both of the tests control the type I error rates well in testing for haplotype-based HWE. If the NM model is true, then LRT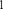 is more powerful. While, if the true model is the IM model, then LRT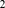 has better performance in power. Under the population stratification model, LRT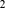 is still more powerful. To this end, LRT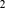 is generally recommended. Application of the proposed methods to a rheumatoid arthritis data set further illustrates their utility for real data analysis.

## Introduction

In studies of genetic epidemiology, complex diseases are often associated with multiple (interacting) markers [1]–[3]. As such, haplotype-based analysis has gained increasing attention as it can potentially be more efficient than a single-marker-based analysis [4]–[9]. Therefore, haplotype inference of unphased genotypes may be expected to play an important role in disease fine mapping [10]. Nowadays, there are many statistical and computational methods available for inferring haplotypes based on different types of data, such as unrelated individuals. One of the popular approaches is the likelihood method, and the maximum likelihood estimation via the expectation-maximization (EM) algorithm [11] is a frequently employed method for haplotype inference. For genotype data of unrelated individuals, an EM-based maximum likelihood method for the estimation of haplotype frequencies was first proposed by Excoffier and Slatkin [12]. We call it EM algorithm in this paper for easy description later. However, the EM algorithm needs the assumption that the population under study is in Hardy-Weinberg equilibrium (HWE), otherwise the estimates of haplotype frequencies may be biased.

Recently, there have been many case-control studies proposed to test for association between haplotypes and disease. The likelihood ratio test (LRT) was constructed from the maximum likelihood functions for cases, controls and the pooled data of cases and controls, to test for haplotype-disease association, which requires the assumption of HWE in the pooled sample data [3]. Prospective likelihood methods based on logistic regression or generalized linear models were investigated by Schaid et al. [13], Stram et al. [14], Zaykin et al. [15], and others. These methods treat unobserved haplotypes as covariates in a regression model and compute the conditional expectation of the covariates given genotype observations under the null hypothesis of no association with a HWE assumption in the pooled sample of cases and controls. Zhao et al. [16] proposed a prospective estimating-equation approach for the assessment of disease association with haplotypes when adjustment for covariates, which needs the HWE assumption of haplotype frequencies only in the control sample. The pooled sample of cases and controls is not necessarily in HWE. On the other hand, a retrospective likelihood method can be used in detecting haplotype-disease association in a case-control study and also requires HWE only in the control population [17]. Therefore, the detection of haplotype-based HWE is crucial prior to fine mapping and positional cloning studies for case-control designs.

The goodness-of-fit test is a frequently-used method to test for HWE for multiple tightly-linked loci. However, when the number of loci under study increases, the degrees of freedom dramatically increase, which may lack the test power. As such, in this paper, to investigate more powerful haplotype-based HWE tests, we first recall three models which can cause Hardy-Weinberg disequilibrium (HWD). One was proposed originally by Niu et al. [6], which includes a parameter 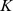 and is called Niu's model (NM) in this paper for convenience; the second one is the inbreeding model (IM) with incorporating the inbreeding coefficient 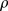 [18]; the third one is a population stratification (PS) model, which can also lead to HWD. Then, we write out two likelihood functions of the observed data based on the NM and IM models, respectively. We develop an expectation-conditional-maximization (ECM) algorithm [19] for the NM model to estimate the parameter 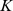 and haplotype frequencies and suggest an EM algorithm for the IM model (denoted by IEM algorithm here) to estimate the inbreeding coefficient 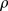 and haplotype frequencies. Note that 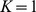 or 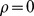 means that HWE holds. So, we further propose two LRT tests LRT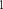 and LRT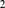 to test for haplotype-based HWE under the NM and IM models, respectively. We simulate the HWE, Niu's, inbreeding and population stratification models to assess the validity and compare the performance of these two LRT tests. The simulation results show that both of the tests control the size well in testing for haplotype-based HWE. If the Niu's model is true, then LRT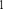 is more powerful. While, if the inbreeding model is true, then LRT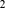 has better performance in power. Under the population stratification model, LRT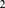 is still more powerful. Therefore, LRT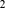 is generally recommended. In addition, we obtain the sum of absolute differences (SAD) between the true and estimated haplotype frequencies [20], and compare the performance of the EM, ECM and IEM algorithms in estimating the haplotype frequencies. If the true model is the Niu's model, then the ECM algorithm has more accurate estimates of haplotype frequencies than the EM and IEM estimates. However, for all the other simulation settings, the EM algorithm is not so much affected by the departure from HWE, and the EM and IEM algorithms almost have the same performance in controlling SAD, which is less than the ECM estimates. Application of the proposed methods to the Rheumatoid Arthritis (RA) data set from the North American Rheumatoid Arthritis Consortium (NARAC) further illustrates their utility for real data analysis.

## Materials and Methods

### Likelihood Function and EM Algorithm under HWE

Consider a sample of 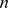 unrelated individuals and 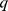 single nucleotide polymorphism (SNP) markers. Assume that the SNPs are tightly linked so that the recombination fraction between any SNP pair is zero. For each SNP, there are two alleles 1 and 2. Let 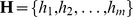 be the set of all possible haplotypes at these 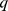 loci, where 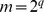. We assume that 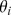 is the frequency of haplotype 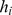 (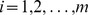), so the set of haplotype frequencies can be denoted by 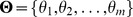. Let 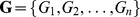 be the set of the observed genotypes of all the 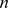 individuals, where 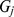 is the genotype of the 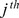 individual. For the 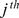 individual, the number of haplotype combinations compatible with 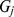 is 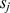. Therefore, the likelihood function of the sample can be expressed as
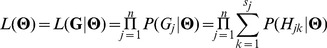
(1)where 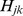 denotes the 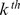 haplotype combination compatible with genotype 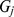 for the 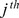 individual.

To make the haplotype frequency estimation easy and feasible, the EM algorithm was employed [11]. Let 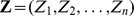 be the true haplotype combinations of the sample which are actually unobserved, and 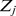 is the true haplotype combination of the 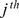 individual. Then the log-likelihood function of the complete data is
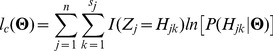
(2)where 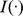 is an indicator function and 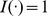 if 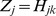 and 0 otherwise. Note that under HWE, the probability 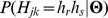 of unordered haplotype pair 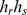 is 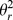 if 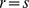 and 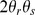 otherwise. Further, Excoffier and Slatkin [12] proposed the following EM algorithm to obtain the maximum likelihood estimates of 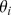 (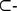) at iteration 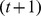,
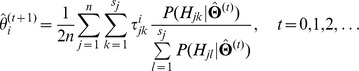
where 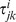 is the number of times that haplotype 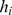 occurs in the 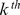 haplotype combination for the 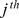 individual and takes values of 0, 1 or 2, and 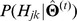 is the value of the probability 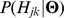 based on the estimated haplotype frequencies 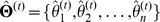 at iteration 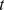.

### Two Forms of HWD

Note that the underlying assumption of HWE is strong and HWE does not hold usually. One may consider the following form of HWD,
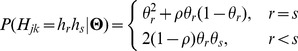
(3)where 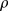 is the inbreeding coefficient which is generally positive [21]. Note that Equation (3) is reduced to HWE when 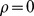. We denote this form of HWD as “inbreeding model (IM)” for convenient description in this paper.

Another form of the departure from HWE was originally proposed by Niu et al. [6] as follows. Assume that the probability of unordered haplotype pair 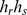 is proportional to 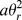 if 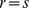 and 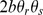 otherwise, with two parameters 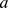 and 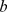. Obviously, the HWE assumption holds if 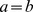. Note that the sum of all these terms for all the 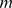 haplotypes at the 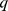 loci may not be 1. Then, HWD can be defined as the following form:
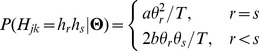
(4)where
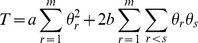



Let 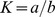. Then, we assume 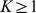 due to the positive inbreeding coefficient 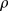. We denote this form of HWD as “Niu's model (NM)” for convenience.

### Likelihood Function and Haplotype-Based HWE Test under Niu's Model

Using Equations (2) and (4), the log-likelihood function of the complete data under the Niu's model can be expressed as
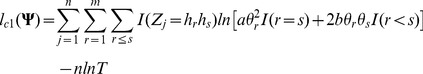
(5)where 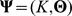. In fact, there is only one additional parameter 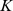 included in Equation (5), compared to the likelihood function under HWE. So, we propose the following expectation-conditional-maximization (ECM) algorithm to estimate the haplotype frequencies and the parameter 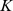. It consists of one expectation step (E-step) and 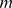 conditional-maximization steps (CM-steps) at each iteration. In E-step at iteration 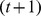, we can get the following 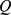 function after taking the conditional expectation of Equation (5), given the observed genotype data 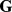 and current estimate 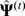 of 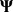,
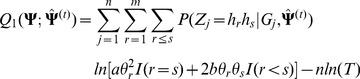
(6)where 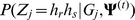 is the conditional probability of the haplotype pair 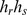 given 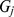 and 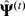, which is 0 if there is no haplotype pair compatible with genotype 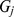.

In CM-steps, we maximize the 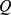 function in Equation (6) to estimate 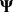. Let 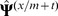 be the estimate of 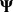 in the 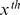 CM-step among 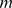 CM-steps at iteration 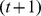. The detailed CM-steps are as follows:

• Give the initial value 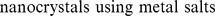, where 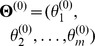.

• At iteration 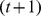, by fixing 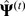 in the first CM-step,   maximize the 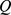 function by taking the first-order derivation   with respect to 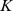 so as to get the estimate of 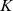, and then
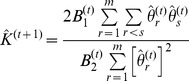
where 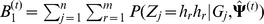, 
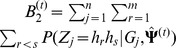
. So, 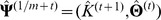.

• Note that there is a constraint condition 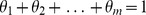  when we maximize 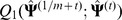 to estimate the haplotype  frequencies 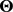. Thus, from the second CM-step to the 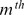 CM- step, 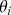
's (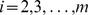) are estimated step by step and 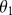 is then  estimated by 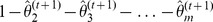. Let 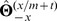 be  the set of the haplotype frequency estimates for all the  haplotypes but 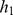 and 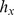 in the 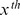 CM-step. Then, 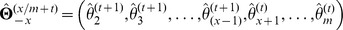. For exam ple, 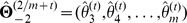 in the second CM-step for estimating 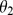. As such, in the 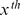 CM-step (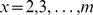), by maximizing 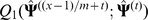, it is shown in Text S1 that a cubic equation with respect to 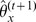 is obtained,
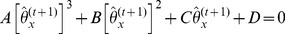
(7)where the coefficients 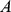, 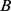, 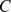 and 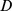 are, respectively,
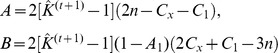


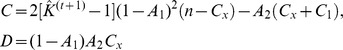


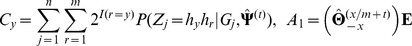


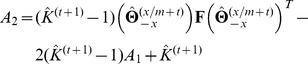
and the vector 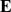 and the matrix 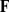 are respectively
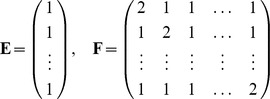



Moreover, the cubic equation above is alway solvable, and its solution can be obtained by Shengjin's formulas [22]. Note that the likelihood function converges no matter which initial values of 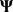 are chosen. So, if there are two or three solutions between 0 and 1, then we can choose the solution which is closer to 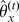 in the former step. After this step,
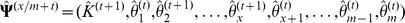



• For 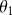, 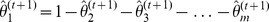. Then 
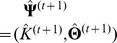
.

• Repeat the steps above until the observed log-likelihood   function of Equation (1) converges.

Equation (1) can be written to be 
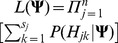
 under the Niu's Model. Note that HWE holds when 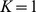 and HWE is violated otherwise. Therefore, a likelihood ratio test (LRT) for HWE is naturally constructed based on the estimated haplotype frequencies as follows,
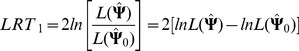
(8)where 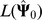 and 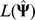 are the values of the observed likelihood function under the null hypothesis of HWE and under the HWD alternative, respectively. Obviously, this LRT statistic asymptotically follows a Chi-square distribution with the degree of freedom being 1 when HWE holds.

### Likelihood Function and Haplotype-Based HWE Test under Inbreeding Model

Borrowing the idea of Zeng and Lin on how to estimate the haplotype frequencies based on case-control data for testing for association [18], here we rewrite the likelihood function for unrelated individuals under study and then propose a haplotype-based HWE test under the inbreeding model. Let 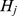 be a random variable, which takes values from 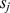 possible haplotype combinations compatible with 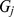 of the 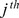 individual. Suppose that 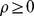, and 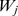 is a Bernoulli variable with success probability 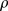. Let 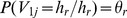 and 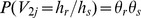, where 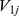 and 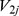 are discrete random variables, and the haplotype before “/” is paternal and haplotype after “/” is maternal. So, 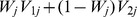 has the same distribution as 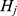, and we treat 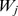, 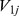 and 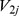 as missing. Then, the log-likelihood function of the complete data under the inbreeding model is
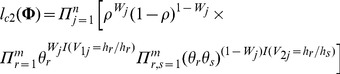
(9)where 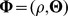.

To estimate the parameters 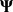 in Equation (9), the EM algorithm is considered. In E-step, the 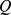 function is
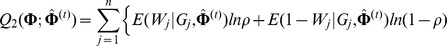


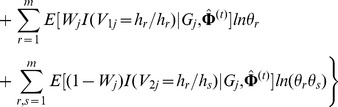



In M-step, the estimation of 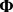 at iteration 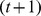 can be obtained by solving the following equation
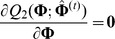



So, 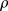 can be estimated by
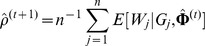


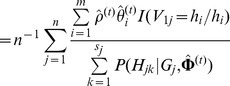
where 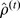 and 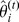 are the estimates of 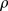 and 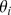 at iteration 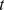, respectively. The haplotype frequencies can be estimated by
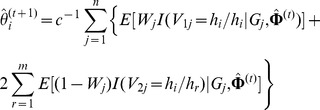
where 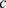 is a normalizing constant, and 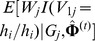 and 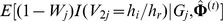 can be calculated as follows,
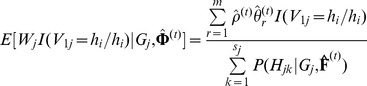


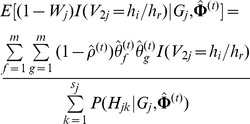



We call this process IEM algorithm for distinguishing it from the previous EM algorithm under HWE.

Note that under the IM model, HWE holds when 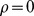, and HWE is not true when 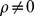. Therefore, we propose the following LRT to test for haplotype-based HWE,
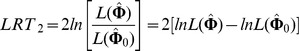
where 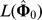 and 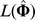 are the values of the observed likelihood function under the null hypothesis of HWE and under the HWD alternative, respectively. Obviously, this LRT statistic asymptotically follows a Chi-square distribution with the degree of freedom being 1 when HWE holds.

### Software Implementation

Based on the above EM, ECM and IEM algorithms, we have written a software HAP-HWE to conduct the proposed haplotype-based HWE tests, which is implemented in R (http://www.r-project.org) and is freely available at http://www.echobelt.org/web/UploadFiles/HAP-HWE.html. For each of the EM, ECM and IEM algorithms, let 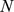 denote the number of haplotypes that occur in all the possible haplotype combinations compatible with the observed genotypes 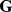 in the sample. As such, the initial values of all these 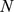 haplotype frequencies are taken as 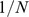 at 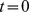. For the ECM and IEM algorithms, the initial values of 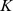 and 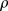 are taken as 1 and 0.01, respectively. The convergence criterion is that the absolute difference between the estimated values of the log-likelihood function at two consecutive iterations is smaller than 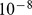. The default maximum number of iterations is 1000. Then, the last estimates, 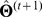, 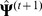 and 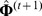, are taken as the maximum likelihood estimates of 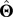, 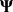 and 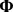, respectively. Consequently, the values of LRT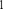 and LRT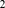 and the corresponding P values are obtained.

The input data file is a standard linkage pedigree file containing pedigree relationship, genotype and phenotype information, with each row being for an individual. The HAP-HWE software will only use the founders in the sample and automatically exclude the nonfounders from the analysis. Further, a haplotype block file is needed with each row representing a haplotype block, which can be easily exported from other existing software, such as Haploview [23]. Then, our HAP-HWE software will analyze the haplotype blocks one by one. The usage of the HAP-HWE software and other details refer to Text S2.

Our HAP-HWE software outputs: (i) the convergence processes of the log-likelihood function under the EM, ECM and IEM algorithms, (ii) the haplotypes with frequency estimates being larger than 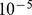 and the associated frequency estimates under the three algorithms, (iii) the estimated value of 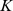, the value of LRT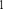 and the corresponding P value under the Niu's model, and (iv) the estimated value of 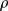, the value of LRT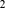 and the corresponding P value under the inbreeding model. The output results will be saved in a text file (named “results.txt”) in the working directory. In addition, like other haplotype frequency estimation methods, our methods also face running time and storage space problems because of the large number of possible haplotypes. In our software, to reduce storage space, each haplotype is represented by an integer, rather than a vector of alleles.

## Results

### Simulation Settings

To assess the validity and compare the performance of two LRT tests in testing for haplotype-based HWE, we consider three models with three tightly-linked SNPs that can lead to HWD: Niu's model (NM), inbreeding model (IM) and population stratification (PS) model. For both the NM and IM models, the true marginal haplotype distribution is given in Table 1. For the NM model, the value of 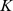 is taken from 1.0 to 1.5 in increments of 0.05. Firstly, we calculate the probabilities of all the haplotype combinations from Equation (4). Then, one haplotype combination for each individual is randomly chosen. For the IM model, the inbreeding coefficient 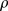 is taken from 0 to 0.1 in increments of 0.01. Firstly, we calculate the probabilities of all the haplotype combinations from Equation (3), and then one haplotype combination is selected at random for each individual. Finally, we combine these two haplotypes to form the unphased genotype for the individual. To investigate how the population admixture affects the performance of two haplotype-based HWE tests, we consider the following PS model with two subpopulations I and II, where the corresponding haplotype distributions are given in Table 2, respectively. The proportion 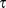 of the subpopulation I is taken to be 0.6 and 0.8.

**Table 1:** Haplotype distribution for Niu's model and inbreeding model.

SNP	Frequency
122	0.082
221	0.525
121	0.283
211	0.004
111	0.106

**Table 2:** Haplotype distribution for population stratification model.

SNP	Frequency	
	I	II
122	0.082	0.030
212	0.000	0.170
112	0.000	0.050
221	0.525	0.470
121	0.283	0.100
211	0.004	0.150
111	0.106	0.030

Note that when 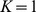 and 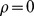, HWE holds for the NM and IM models, respectively. So, we simulate the type I error rates of the proposed HWE tests when 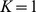 or 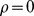, and make power comparison when 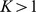 and 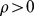. The PS model is also used to simulate the powers of both of the tests. For all the models, we generate samples of unrelated individuals at these three loci and the sample size is taken as 500, 1000 and 1500, respectively. The number of simulation replicates is fixed at 1000 and the significance level 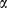 is taken to be 5%.

As additional findings in this paper, we can also compare the efficiency of the EM, ECM and IEM algorithms in haplotype inference. The accuracy of haplotype frequency estimates is assessed by the sum of absolute differences (SAD) between the true and estimated frequencies, which was proposed by Fallin and Schork [20] and defined as
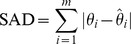
where 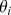 and 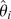 are the true and estimated haplotype frequencies of 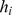, respectively. It ranges from 0 (when the estimation is perfect) to 1.

### Simulation Results

Table 3 lists the estimate of 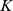, mean SAD of haplotype frequency estimates, simulated size and powers of two HWE tests for different values of 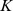 and different sample sizes 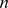 under the Niu's model. It is shown in the table that the mean estimated value 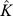 over 1000 replicates is close to its true value. The type I error rate of LRT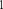 is close to the nominal 5% level, while the size result of LRT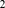 is less than 0.05, when 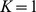 (i.e. HWE holds). This means that in testing for haplotype-based HWE, LRT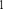 controls the size well and LRT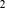 is conservative under the NM model. The powers of both LRT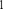 and LRT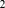 are larger when 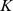 increases from 1.1 to 1.5 and the sample size 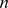 is fixed. However, LRT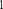 is more powerful than LRT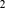. In addition, when 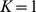 and 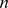 is unchanged, the EM, ECM and IEM algorithms perform similarly in the estimation of haplotype frequencies. However, with the increase of the 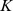 value, the SAD measure of the ECM algorithm does not have much change and is much smaller than the EM and IEM algorithms. The SADs of the EM and IEM algorithms are very close to each other and become larger when 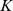 is larger. On the other hand, with the sample size increasing, the SAD measures of all the three algorithms become less and two proposed LRT tests have more powers.

**Table 3:** Mean and standard deviation (SD) of 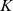 and 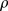 estimates, mean of sum of absolute differences (SAD) of haplotype frequency estimates for EM, ECM and IEM algorithms, simulated size and powers of two HWE tests for different values of 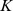 and 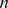, under Niu's model.

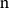	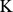	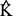		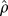		SAD			Size/Power	
		Mean	SD	Mean	SD	EM	ECM	IEM	LRT 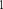	LRT 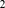
500	1.00	1.001	0.107	0.009	0.016	0.041	0.043	0.041	0.054	0.029
	1.05	1.055	0.115	0.016	0.020	0.041	0.043	0.041	0.072	0.066
	1.10	1.099	0.118	0.022	0.022	0.043	0.043	0.043	0.135	0.107
	1.15	1.148	0.123	0.030	0.024	0.048	0.045	0.048	0.240	0.174
	1.20	1.198	0.124	0.039	0.027	0.050	0.043	0.050	0.368	0.274
	1.25	1.241	0.132	0.048	0.028	0.053	0.043	0.053	0.528	0.397
	1.30	1.299	0.138	0.060	0.030	0.058	0.044	0.058	0.672	0.543
	1.35	1.346	0.136	0.070	0.030	0.061	0.043	0.060	0.799	0.678
	1.40	1.396	0.144	0.079	0.031	0.068	0.043	0.067	0.880	0.776
	1.45	1.454	0.150	0.090	0.031	0.071	0.043	0.070	0.933	0.860
	1.50	1.497	0.158	0.099	0.033	0.075	0.042	0.073	0.966	0.899
1000	1.00	1.001	0.076	0.007	0.010	0.029	0.031	0.029	0.050	0.019
	1.05	1.047	0.080	0.013	0.014	0.030	0.031	0.030	0.097	0.073
	1.10	1.096	0.082	0.020	0.017	0.033	0.031	0.033	0.229	0.157
	1.15	1.148	0.085	0.030	0.019	0.036	0.030	0.036	0.452	0.329
	1.20	1.201	0.089	0.040	0.020	0.041	0.031	0.040	0.673	0.532
	1.25	1.247	0.091	0.049	0.021	0.045	0.030	0.044	0.839	0.695
	1.30	1.296	0.094	0.059	0.021	0.050	0.030	0.049	0.933	0.838
	1.35	1.352	0.101	0.071	0.022	0.055	0.030	0.054	0.975	0.920
	1.40	1.398	0.106	0.080	0.023	0.060	0.031	0.058	0.985	0.961
	1.45	1.444	0.104	0.090	0.022	0.066	0.031	0.064	0.996	0.987
	1.50	1.504	0.111	0.101	0.023	0.070	0.030	0.068	1.000	0.999
1500	1.00	1.001	0.060	0.005	0.008	0.024	0.026	0.024	0.039	0.023
	1.05	1.047	0.063	0.011	0.012	0.025	0.025	0.025	0.111	0.082
	1.10	1.102	0.070	0.021	0.015	0.028	0.025	0.028	0.347	0.267
	1.15	1.148	0.072	0.029	0.016	0.031	0.025	0.031	0.611	0.464
	1.20	1.199	0.073	0.040	0.017	0.036	0.025	0.036	0.841	0.690
	1.25	1.249	0.073	0.050	0.017	0.042	0.025	0.041	0.958	0.865
	1.30	1.302	0.080	0.061	0.018	0.047	0.025	0.047	0.990	0.950
	1.35	1.349	0.081	0.070	0.018	0.053	0.025	0.051	0.999	0.984
	1.40	1.403	0.083	0.081	0.018	0.058	0.025	0.056	1.000	0.994
	1.45	1.449	0.087	0.091	0.018	0.064	0.025	0.062	1.000	0.998
	1.50	1.499	0.086	0.100	0.018	0.068	0.025	0.066	1.000	1.000

Table 4 shows the estimate of 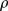, mean SAD of haplotype frequency estimates, simulated size and powers of two HWE tests for different values of inbreeding coefficient 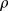 and different sample sizes 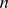 under the inbreeding model. We can see from the table that the mean estimated value 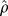 over 1000 replicates is close to its true value. As shown in Table 3, LRT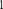 performs better in controlling the size than LRT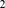 under the IM model. However, LRT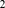 is more powerful than LRT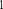 under this situation. On the other hand, both the EM and IEM algorithms have the same performance and the corresponding SADs are stable across different values taken for 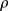 (0 to 0.1) in the estimation of haplotype frequencies. However, the ECM estimate gets larger with the increase of 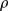 and performs worse than the EM and IEM estimates. When the sample size is larger, the corresponding SADs appear to be smaller and two proposed LRT tests are more powerful.

**Table 4:** Mean and standard deviation (SD) of 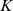 and 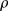 estimates, mean of sum of absolute differences (SAD) of haplotype frequency estimates for EM, ECM and IEM algorithms, simulated size and powers of two HWE tests for different values of 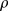 and 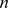, under inbreeding model.

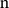	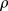	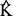		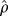		SAD			Size/Power	
		Mean	SD	Mean	SD	EM	ECM	IEM	LRT 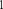	LRT 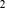
500	0.00	1.001	0.111	0.010	0.015	0.041	0.044	0.041	0.060	0.020
	0.01	1.035	0.108	0.015	0.019	0.041	0.044	0.041	0.048	0.060
	0.02	1.077	0.120	0.024	0.023	0.041	0.044	0.041	0.114	0.137
	0.03	1.109	0.123	0.030	0.025	0.042	0.047	0.042	0.170	0.218
	0.04	1.157	0.124	0.041	0.027	0.041	0.048	0.041	0.268	0.345
	0.05	1.185	0.124	0.049	0.028	0.042	0.049	0.042	0.357	0.468
	0.06	1.241	0.131	0.061	0.028	0.041	0.051	0.041	0.528	0.629
	0.07	1.279	0.141	0.070	0.030	0.042	0.054	0.042	0.638	0.735
	0.08	1.316	0.144	0.079	0.031	0.043	0.057	0.043	0.718	0.811
	0.09	1.366	0.140	0.090	0.029	0.044	0.061	0.043	0.851	0.906
	0.10	1.409	0.156	0.099	0.032	0.043	0.064	0.043	0.889	0.940
1000	0.00	1.000	0.075	0.007	0.010	0.029	0.031	0.029	0.056	0.026
	0.01	1.032	0.076	0.012	0.014	0.029	0.030	0.029	0.053	0.077
	0.02	1.073	0.078	0.021	0.016	0.029	0.032	0.029	0.142	0.198
	0.03	1.115	0.084	0.030	0.018	0.029	0.034	0.029	0.294	0.394
	0.04	1.151	0.086	0.040	0.019	0.030	0.037	0.030	0.477	0.606
	0.05	1.188	0.088	0.049	0.020	0.030	0.039	0.030	0.643	0.762
	0.06	1.229	0.093	0.059	0.020	0.031	0.042	0.031	0.775	0.875
	0.07	1.276	0.097	0.070	0.021	0.029	0.045	0.029	0.895	0.957
	0.08	1.316	0.096	0.079	0.020	0.030	0.048	0.030	0.959	0.988
	0.09	1.357	0.102	0.089	0.021	0.030	0.052	0.030	0.973	0.995
	0.10	1.411	0.102	0.100	0.021	0.031	0.057	0.031	0.996	1.000
1500	0.00	1.001	0.064	0.006	0.009	0.024	0.025	0.024	0.053	0.028
	0.01	1.038	0.062	0.012	0.012	0.024	0.026	0.024	0.079	0.096
	0.02	1.073	0.066	0.020	0.014	0.024	0.027	0.024	0.210	0.272
	0.03	1.111	0.072	0.030	0.016	0.024	0.029	0.024	0.392	0.534
	0.04	1.150	0.070	0.040	0.015	0.024	0.032	0.024	0.610	0.763
	0.05	1.191	0.071	0.050	0.016	0.024	0.035	0.024	0.794	0.902
	0.06	1.231	0.075	0.059	0.017	0.025	0.038	0.025	0.916	0.970
	0.07	1.276	0.082	0.070	0.017	0.024	0.042	0.024	0.975	0.992
	0.08	1.318	0.081	0.080	0.017	0.025	0.047	0.025	0.995	0.997
	0.09	1.365	0.081	0.090	0.017	0.025	0.050	0.025	0.999	0.999
	0.10	1.408	0.086	0.100	0.018	0.025	0.054	0.025	1.000	1.000

Table 5 displays the mean SAD of haplotype frequency estimates and simulated powers of two HWE tests based on 1000 simulation replicates, under the PS model, with the proportion 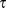 of subpopulation I being taken as 0.6 and 0.8, and the sample size being fixed at 500, 1000 and 1500. From the table, we find that LRT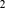 is more powerful than LRT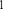, irrespective of the 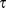 value or the sample size 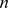. In the estimation of haplotype frequencies, the EM and IEM algorithms perform similarly in SAD and have better SADs than the ECM estimate, which signifies that the EM and IEM algorithm are more robust to population stratification than the ECM algorithm.

**Table 5:** Mean and standard deviation (SD) of 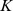 and 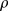 estimates, mean of sum of absolute differences (SAD) of haplotype frequency estimates for EM, ECM and IEM algorithms, power comparison of two HWE tests under population stratification model, with the proportion 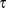 of subpopulation I being taken as 0.6 and 0.8, and the sample size being fixed at 500, 1000 and 1500.

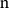	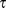	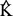		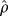		SAD			Power	
		Mean	SD	Mean	SD	EM	ECM	IEM	LRT 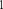	LRT 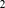
500	0.6	1.224	0.144	0.044	0.022	0.073	0.094	0.072	0.396	0.572
	0.8	1.138	0.131	0.041	0.023	0.061	0.072	0.060	0.197	0.541
1000	0.6	1.223	0.103	0.045	0.016	0.061	0.086	0.061	0.674	0.874
	0.8	1.138	0.092	0.041	0.016	0.047	0.060	0.046	0.350	0.828
1500	0.6	1.226	0.083	0.045	0.013	0.057	0.084	0.056	0.850	0.962
	0.8	1.133	0.076	0.041	0.013	0.042	0.056	0.041	0.463	0.946

### Application to NARAC Data Set

We apply our HAP-HWE software to the Rheumatoid Arthritis (RA) data set from the North American Rheumatoid Arthritis Consortium (NARAC) [24], which was made available through the Genetic Analysis Workshop 15 [25]. In the data set, there are 757 pedigrees comprised of 8017 individuals (2481 founders and 5536 nonfounders), which were genotyped at 5407 SNP markers over the 22 autosomes. In each pedigree, there is at least one affected nonfounder with RA.

Note that information on haplotype blocks is needed prior to the HAP-HWE analysis. In this application, we use the existing software Haploview (version 4.2) [23] to define haplotype blocks, with all the arguments being taken as the default values. Then, 181 haplotype blocks are identified, 150 blocks including 2 SNPs, 19 blocks including 3 SNPs, 7 blocks including 4 SNPs, 1 block including 5 SNPs, 2 blocks including 6 SNPs, 1 block including 9 SNPs and 1 block including 13 SNPs.

On the other hand, HAP-HWE only uses the founders and excludes the nonfounders from the analysis. Further, there is a large proportion of missing genotypes for individuals in the data set. Therefore, the reduced data set used for the HAP-HWE analysis contains only a few founders in the data set. On the average, there are about 295 pedigrees (about 367 unrelated individuals) used for each haplotype block, ranging from 288 to 296 (ranging from 358 to 369).

**Figure 1:**
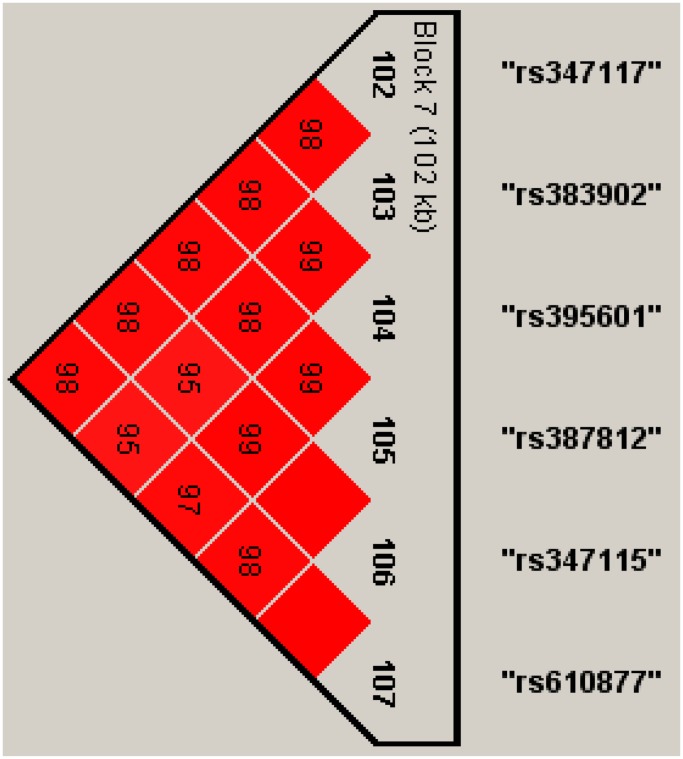
Haplotype LD display for the seventh haplotype block on chromosome 15. The red box denotes that the LOD value between any two loci is larger than or equal to 2.0. The numbers in the red boxes are the corresponding values of 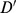 and the empty box denotes that 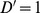.

Table 6 lists the results of the application to the NARAC data set. The significance level is fixed at 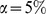. There are 13 haplotype blocks (out of 181) with at least one of the P values of the LRT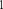 and LRT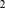 being less than 5%. However, after multiple testing based on Bonferroni correction (
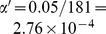
), only the seventh haplotype block including 6 SNPs (rs347117, rs383902, rs395601, rs387812, rs347115 and rs610877) on chromosome 15 is statistically significant with the P value of the LRT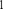 being 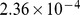. Figure 1 gives the Haploview LD display for this haplotype block. On the other hand, Min et al. [26] reported that chromosome 15p34 at rs347117 showed a possible linkage peak to RA by using the nonparametric linkage 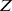 score (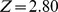), which may support our finding.

**Table 6:** Results of application to North American Rheumatoid Arthritis Consortium data set.

	Haplotype	N. of					
Chr.	block	SNPs	SNP names	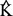	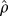	P-value	
						LRT 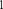	LRT 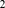
2	3	2	rs1686430, rs1734449	1.349	0.137	0.0107	0.0089
2	9	2	rs1866209, rs1438048	1.563	0.150	0.0051	0.0057
3	1	2	rs1516337, rs1516350	1.287	0.086	0.0204	0.0213
5	6	2	rs244903, rs244896	1.052	0.067	0.6341	0.0311
6	7	2	rs1565528, rs1491074	1.284	0.074	0.0231	0.0505
7	16	2	rs1182378, rs1182414	1.137	0.071	0.2526	0.0440
10	3	2	rs1979720, rs1494201	1.302	0.082	0.0189	0.0313
13	2	2	rs436227, rs390704	1.350	0.096	0.0085	0.0173
14	3	2	rs1381641, rs1020897	1.264	0.077	0.0326	0.0704
15	7	6	rs347117, rs383902,	1.547	0.099	2.36 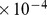	0.0428
			rs395601, rs387812,				
			rs347115, rs610877				
16	5	2	rs179209, rs179219	1.215	0.086	0.0708	0.0388
18	6	2	rs1787190, rs1981	1.330	0.139	0.0070	0.0076
21	3	2	rs1892687, rs2051179	1.372	0.146	0.0027	0.0048

Chr., chromosome; SNP, single nucleotide polymorphism; N. of SNPs, number of SNPs.

## Discussion

In this paper, we first wrote out two likelihood functions of the observed data based on the NM model and IM model. Then, we developed the ECM algorithm for the NM model to estimate the parameter 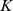 and haplotype frequencies and suggested the IEM algorithm for the IM model to estimate the inbreeding coefficient 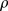 and haplotype frequencies. Note that 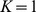 or 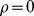 means that HWE holds. So, we further proposed two LRT tests to test for haplotype-based HWE. We simulated the HWE, Niu's, inbreeding and population stratification models to assess the validity and compare the performance of these two LRT tests. The simulation results showed that both of the two tests are valid in testing for the haplotype-based HWE. If the Niu's model is true, then LRT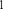 is more powerful. While, if the inbreeding model is true, then LRT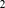 has better performance in power. Under the population stratification model, LRT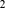 is still more powerful. Therefore, if the population model is unknown in practice, LRT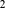 is generally recommended due to its good performance. Furthermore, we compared the performance of the EM, ECM and IEM algorithms in estimating the haplotype frequencies. If the true model is the Niu's model, then the ECM algorithm has more accurate estimates of haplotype frequencies than the EM and IEM estimates. However, for all the other simulation settings, the EM algorithm is not so much affected by the departure from HWE, and the EM and IEM algorithms almost have the same performance in controlling SAD, which is less than the ECM estimates. We also demonstrate the practical utility of the proposed methods by the application to the Rheumatoid Arthritis (RA) data set from the North American Rheumatoid Arthritis Consortium (NARAC). In addition, note that there are many abbreviations and notations used in this paper. So, in Supporting Information, we give two tables (Tables S1 and S2) to list them for the easy reference.

## Supporting Information

Table S1 Summary of abbreviations.
(PDF)

Table S2 Summary of notations.
(PDF)

Text S1 Conditional-maximization steps of ECM algorithm.
(PDF)

Text S2 Help file of HAP-HWE.
(PDF)
