# Supplementary material for: Powerful Haplotype-Based Hardy-Weinberg Equilibrium Tests for Tightly Linked Loci
Source: PLoS One. 2013 Oct 22;8(10):e77399. doi: 10.1371/journal.pone.0077399 (PMC3805574; doi:10.1371/journal.pone.0077399)
Supplement: Table S1 — Summary of abbreviations. (PDF) [file pone.0077399.s001.pdf]

Table S1. Summary of abbreviations.

**Abbreviation**	**Full name**
CM-step	conditional-maximization step
ECM	expectation-conditional-maximization algorithm
EM	expectation-maximization algorithm
E-step	expectation step
HWD	Hardy-Weinberg disequilibrium
HWE	Hardy-Weinberg equilibrium
IEM	expectation-maximization algorithm for inbreeding model
IM	inbreeding model
LRT	likelihood ratio test
M-step	maximization step
NARAC	North American Rheumatoid Arthritis Consortium
NM	Niu's model
PS	population stratification model
RA	rheumatoid arthritis
SAD	sum of absolute differences
SNP	single nucleotide polymorphism
