# Supplementary material for: Powerful Haplotype-Based Hardy-Weinberg Equilibrium Tests for Tightly Linked Loci
Source: PLoS One. 2013 Oct 22;8(10):e77399. doi: 10.1371/journal.pone.0077399 (PMC3805574; doi:10.1371/journal.pone.0077399)
Supplement: Table S2 — Summary of notations. (PDF) [file pone.0077399.s002.pdf]

Table S2. Summary of notations.

Notation	Meaning
θ_i	single haplotype frequency
$\mathbf{\Theta}$	set of haplotype frequencies
ρ	inbreeding coefficient
$\mathbf{\Phi}$	$\mathbf{\Phi} = (\rho, \mathbf{\Theta})$
$\mathbf{\Psi}$	$\mathbf{\Psi} = (K, \mathbf{\Theta})$
G_j	single genotype
$\mathbf{G}$	set of genotypes
h_i	single haplotype
H_{jk}	single haplotype combination compatible with G_j
$\mathbf{H}$	set of haplotypes
i, r, s, f, g	indexing haplotype or haplotype frequency
j	indexing individual
K	$K = a/b$, which is a parameter used in Niu's model
k, l	indexing haplotype combination
m	number of all possible haplotypes at q loci
n	sample size
q	number of loci
s_j	number of haplotype combinations compatible with G_j
t	indexing iteration
x	indexing CM-step
Z_j	true haplotype combination of individual j
$\mathbf{Z}$	set of true haplotype combinations of the sample
