# Supplementary material for: Powerful Haplotype-Based Hardy-Weinberg Equilibrium Tests for Tightly Linked Loci
Source: PLoS One. 2013 Oct 22;8(10):e77399. doi: 10.1371/journal.pone.0077399 (PMC3805574; doi:10.1371/journal.pone.0077399)
Supplement: Text S1 — Conditional-maximization steps of ECM algorithm. (PDF) [file pone.0077399.s003.pdf]

Text S1 Conditional-maximization steps of ECM algorithm

Take the expectation of $l_{c1}(\mathbf{\Psi})$ with respect to $\mathbf{Z}$, then we have the following Q function,

$$\begin{aligned} Q_1(\mathbf{\Psi};\hat{\mathbf{\Psi}}^{(t)}) &= E_{\mathbf{Z}}[l_{c1}(\mathbf{\Psi})] \\ &= \sum_{j=1}^{n}\sum_{r=1}^{m}\sum_{r<s} E[I(Z_j = h_r h_s)|G_j, \hat{\mathbf{\Psi}}^{(t)}]ln\left[a\theta_r^2 I(r=s) + 2b\theta_r\theta_s I(r<s)\right] - nlnT \\ &\hat{=} R - S \end{aligned}$$

There are $(m+1)$ parameters to estimate (the parameter K and m haplotype frequencies) in the Q function and only one parameter is estimated in each CM-step. Further, note that when we maximize the Q function, there is a constraint condition that the sum of all the haplotype frequencies is equal to 1. So, there are m CM-steps in maximizing the Q function. Here, suppose that θ_1 is calculated by others. Let θ_x $(x = 2, 3, \ldots, m)$ denote the haplotype frequency which will be estimated in the xth CM-step. As such, θ_1 can be estimated by $\hat{\theta}_1 = 1 - \hat{\theta}_2 - \ldots - \hat{\theta}_x - \ldots - \hat{\theta}_m = 1 - \hat{\theta}_x - A_1$. Then

$$\begin{aligned} T &= a\sum_{r=1}^{m}\theta_r^2 + 2b\sum_{r=1}^{m}\sum_{r<s}\theta_r\theta_s \\ &= 2(a-b)\theta_x^2 - 2(a-b)(1-A_1)\theta_x + \Bigg[2(a-b)\sum_{r=2,r\neq x}^{m}\theta_r^2 \\ &\quad +2(a-b)\sum_{r=2,r\neq x}^{m}\sum_{s\neq x,r<s}\theta_r\theta_s - 2(a-b)A_1 + a\Bigg] \end{aligned}$$

To estimate the haplotype frequencies, take the first-order derivation of Q_1 with respect to θ_x and we can get the following equations,

$$\begin{aligned} \frac{\partial Q_1}{\partial \theta_x} &= \frac{\partial R}{\partial \theta_x} - \frac{\partial S}{\partial \theta_x} = 0 \\ \frac{\partial R}{\partial \theta_x} &= \sum_{j=1}^{n}\sum_{r=1}^{m}\frac{2^{I(r=x)}}{\theta_x}P(Z_j = h_x h_r|G_i, \hat{\mathbf{\Psi}}^{(t)}) - \sum_{j=1}^{n}\sum_{r=1}^{m}\frac{2^{I(r=1)}}{\theta_1}P(Z_j = h_1 h_r|G_j, \hat{\mathbf{\Psi}}^{(t)}) \\ \frac{\partial S}{\partial \theta_x} &= \frac{n}{T}\left[4(a-b)\theta_x - 2(a-b)(1-A_1)\right] \end{aligned}$$

Using the equations above, Equation (7) can be obtained.
