# Supplementary material for: Powerful Haplotype-Based Hardy-Weinberg Equilibrium Tests for Tightly Linked Loci
Source: PLoS One. 2013 Oct 22;8(10):e77399. doi: 10.1371/journal.pone.0077399 (PMC3805574; doi:10.1371/journal.pone.0077399)
Supplement: Text S2 — Help file of HAP-HWE. (PDF) [file pone.0077399.s004.pdf]

Text S2 Help file of HAP-HWE

This document contains the instructions for using the HAP-HWE software in R.

1. Usage

Download the R source code “HAP-HWE.txt”. Use

> source(”HAP-HWE.txt”)

at the R command line to read in the HAP-HWE software.

2. Example

Download the sample data file “ped.txt” and the haplotype block file “block.txt”. Then use

> HAPHWE(”ped.txt”, header=T, missing=0, blockfile=”block.txt”, filename=”results.txt”)

at the R command line to analyze the sample data file.

3. Output

The HAP-HWE software outputs the results for haplotype blocks one by one, which will be automatically saved in a text file named “results.txt” in the working directory. For each haplotype block, the HAP-HWE outputs:

(1) the haplotype block name;

(2) the convergence processes of the log-likelihood function under the EM, ECM and IEM algorithms;

(3) the haplotypes with frequency estimates being larger than 10^{-5} and the associated frequency estimates under the three algorithms;

(4) the estimated value of K, the value of LRT_1 and the corresponding P value under the Niu’s model;

(5) the estimated value of ρ, the value of LRT_2 and the corresponding P value under the inbreeding model.

4. Arguments

The arguments in the usage

> HAPHWE(”ped.txt”, header=T, missing=0, blockfile=NULL, filename=”results.txt”, start.ratio.ab = 1, start.rho = 0.01, iter = 1000, dv = 1e-8)

are as follows:

ped: The name of a standard linkage pedigree file or a matrix/dataframe containing pedigree relationship, genotype and phenotype information with each row being for an individual. The first 5 columns give the pedigree id, individual id, father id (0 if founder), mother id (0 if founder) and sex (1=male, 2=female), in that order. Note that these fields need to be numeric. The sixth column gives the affection status (1=unaffected; 2=affected; 0=unknown). The remaining columns code for marker genotypes (2 column per SNP), with two alleles 1 and 2 for each marker. The HAP-HWE software will automatically exclude the nonfounders from the analysis.

header: If the data file has header, then it should be taken as T (or TRUE), otherwise F (or FALSE). The default value is T.

missing: The missing value for alleles. The default value is 0. It could take any numeric value, except for allele codes 1 and 2, and NA, but could not take any string value. If there is any missing values at some loci on a haplotype block for an individual, then the individual will be excluded from the analysis when analyzing this haplotype block.

blockfile: The haplotype block file with each row representing a haplotype block, the first column representing the id of the haplotype block and the columns behind the first column representing the marker sequence numbers included in the haplotype block. For example, see the first row 1 1 2 3 in the sample block file. The first number 1 denotes the first haplotype block; the last three numbers denote that this haplotype block includes the first, second and third SNPs in the sample pedigree data file. Also, see the second row 2 4 5 in the sample block file. The first number 2 denotes the second haplotype block; the last two numbers denote that this haplotype block includes the fourth and fifth SNPs in the sample pedigree data file. Note that there should be at least one blank line at the end of the haplotype block file. If the "blockfile" is assigned, then the HAP-HWE software analyzes the haplotype blocks one by one. Otherwise, the HAP-HWE software will simultaneously analyze all the markers in the input file ("ped.txt") as a whole haplotype block. The default value of the "blockfile" is NULL.

filename: If the "filename" is assigned (should be text file, and the file name should be embraced by double quotation marks, then the output results will be saved in the text file in the working directory. Otherwise, the output results will be automatically saved in the "results.txt" file.

start.ratio.ab: The initial value of the parameter $K = a/b$ under the Niu's model for iterations. The default value is 1.

start.rho: The initial value of the parameter ρ under the inbreeding model for iterations, which should be taken to be larger than 0. The default value is 0.01.

iter: The maximum number of iterations. The default value is 1000.

dv: The convergence criterion. The default value is 10^{-8}.
